# Free Radical Scavenging Properties and Induction of Apoptotic Effects of Fa Fraction Obtained after Proteolysis of Bioactive Peptides from Microalgae *Synechococcus* sp. VDW

**DOI:** 10.17113/ftb.57.03.19.6028

**Published:** 2019-09

**Authors:** Rutairat Suttisuwan, Saranya Phunpruch, Tanatorn Saisavoey, Papassara Sangtanoo, Nuttha Thongchul, Aphichart Karnchanatat

**Affiliations:** 1 Program in Biotechnology, Faculty of Science, Chulalongkorn University, 254 Phayathai Road, Wangmai, Pathumwan, Bangkok 10330, Thailand; 2 Department of Biology, Faculty of Science, King Mongkut’s Institute of Technology Ladkrabang, Chalongkrung Road, Ladkrabang, Bangkok 10520, Thailand; 3 Bioenergy Research Unit, Faculty of Science, King Mongkut’s Institute of Technology Ladkrabang, Chalongkrung Road, Ladkrabang, Bangkok 10520, Thailand; 4 Institute of Biotechnology and Genetic Engineering, Chulalongkorn University, 254 Phayathai Road, Pathumwan, Bangkok 10330, Thailand; 5 Research Unit in Bioconversion/Bioseparation for Value-Added Chemical Production, Institute of Biotechnology and Genetic Engineering, Chulalongkorn University, 254 Phayathai Road, Wangmai, Pathumwan, Bangkok 10330, Thailand

**Keywords:** antioxidant, apoptosis, cancer, protein hydrolysate, *Synechococcus* sp. VDW, SW620 cell line

## Abstract

This study aims to determine the antioxidant activity of bioactive peptides derived from *Synechococcus* sp. VDW cells cultured for 21 days. *Synechococcus* sp. VDW protein hydrolysates were prepared with trypsin and purified by ultrafiltration with molecular mass cut-off membranes of 10, 5 and 3 kDa. The *M*<3 kDa (F_A_) fraction had the highest 2,2’-azino-bis(3-ethylbenzothiazoline-6-sulphonic acid) (ABTS) and 2,2’-diphenyl-1-picrylhydrazyl (DPPH) radical scavenging activities, with IC_50_ values of (11.5±0.3) and (13.6±0.2) µg/mL, respectively. The F_A_ fraction was separated by reversed phase HPLC to yield four subfractions (F_1–4_). The F_4_ subfraction showed the highest maximum ABTS radical scavenging activity (3.55±0.61) % and it was selected for further analysis by electrospray ionisation quadrupole time-of-flight mass spectrometry (ESI-Q-TOF-MS/MS) based on *de novo* peptide sequencing. Five antioxidant peptides were identified, of which AILESYSAGKTK had the highest ABTS radical scavenging activity. Furthermore, the F_A_ fraction showed high cytotoxic activities against human cancer-derived cell lines, especially the colon cancer cell line (SW620) with an IC_50_ value of (106.6±21.5) µg/mL, but not the untransformed Wi38 cell line. The F_A_ fraction activated the apoptotic pathway in SW620 cells after treatment for 24, 48 and 72 h, with the highest activities of caspases-3, -8 and -9 being observed after treatment for 72 h. These findings suggested that microalgae *Synechococcus* sp. VDW may be used to develop natural anticancer drugs.

## INTRODUCTION

Together, more than 100 different types of cancer constitute one of the most life-threatening diseases (*1*). Due to the lack of effective drugs, the expensive cost of chemotherapeutic agents and increase in resistance to them over treatment time, plus the adverse side effects of their systemic administration, along with the frequent asymptomatic nature of the early stages, cancer is often fatal. Developing new effective treatments is necessary to cure cancer with minimal side effects to the patient. The use of bioactive compounds, called biotherapy, is an interesting new method of cancer treatment that is widely studied in various countries (*2*). Cell death can be induced through several different mechanisms, including the most widely described methods of apoptosis and necrosis (*3*).

Free radicals are any species able to independently exist such as atoms or molecule that contain one or more unpaired electrons in the outer orbital. This unpaired electron gives a considerable degree of reactivity to the free radical. The ground state of molecular oxygen has two unpaired electrons in its outer shell. Therefore, a radical species can occur by itself. Uncoupled electrons are very reactive with adjoining molecules such as lipid, proteins and carbohydrates, and can cause cellular damage (*4*). The most biologically important free radicals are the radical derivatives of oxygen known as reactive oxygen species (ROS). The presence of high levels of ROS can be associated with the disturbance of cell function and could lead to apoptosis.

Natural products obtained from microalgae are divided into a diverse range of structural classes, including peptides, polypeptides, alkaloids, lipids and terpenes, whose structures can be complex. Recently, endogenous marine peptides have been used to develop novel pharmaceutical agents (*5*). Therefore, there is an increasing demand to isolate new functional protein or bioactive peptides from microalgae. Over the years, the biological activities of enzyme extracts from microalgae have been studied and found to be based on their inherent amino acid composition and sequence, typically varying from 2–20 amino acid constituents. In addition, peptides from microalgae have become of interest due to their reported specific health benefits (*6*, *7*). Some microalgal species have beneficial biological activities due to the presence of proteins, their hydrolysates or peptides, such as antioxidant, antihypertensive, immunomodulatory and anticancer activities, in addition to their nutritional values. Soy protein has antioxidant properties, and polypeptides from digested soy protein can inhibit ovarian cancer cells by hindering the activity of urokinase (*8*). In the present study, we purified the oligopeptides with the potential anticancer (cytotoxic) activity from the trypsin digestion of the microalgal protein from *Synechococcus* sp. VDW, and then investigated their effects on cell proliferation, apoptotic effects and antioxidant activity. The results of this study can be used to further develop the obtained peptides as substitutes for chemotherapeutic agents which have severe side effects.

## MATERIALS AND METHODS

### Microorganism

Wild-type *Synechococcus* sp. VDW (GenBank database accession number MH393765), collected from Ao Wong Duan, Koh Samet, Thailand in July 2012 (*9*), was selected as the representative microalgal strain in this study. The cultures were grown in BG11 broth combined with Turks Island salt solution (pH=7.5; Merck, Kenilworth, NJ, USA) for maintenance and cell production (*10*) and were incubated at 30 °C in 250-mL flasks under daylight fluorescent tubes with light intensity of 30 µmol/(m^2^·s). Cell growth was followed by monitoring the absorbance at 750 nm. Cells were harvested by centrifugation (centrifuge model Kubota 6500; Shimadzu, Kyoto, Japan) at 9880×g for 15 min, and washed twice with 50 mM potassium phosphate buffer (pH=7.0; Sigma-Aldrich, Merck). They were homogenated with glass beads in extraction buffer (50 mM potassium phosphate buffer (pH=7.0), 0.1 mM EDTA 0.1% (*V*/*V*) (Lonza, Verviers, Belgium), Triton X-100 (Sigma-Aldrich, Merck) and 0.05% (*m*/*V*) polyvinylpyrrolidone-40 (PVP-40; Sigma-Aldrich, Merck) using homogenizer (model UR-21P; Tomy Seiko, Tokyo, Japan). The homogenate was centrifuged (model Kubota 6500; Shimadzu) at 9880*×g* for 15 min. The supernatant fraction was used as crude protein for determination of protein content. All operations were carried out at 4 °C.

### Enzymatic hydrolysis of cell biomass

*Synechococcus* sp. VDW cells were disrupted by sonication with glass beads in potassium phosphate buffer (Sigma-Aldrich, Merck) and cell debris was harvested by centrifugation (model Kubota 6500; Shimadzu) at 9880*×g* for 15 min. The remaining biomass with 20% solid content was dehydrated and used as crude protein. Trypsin from porcine pancreas (Sigma-Aldrich, Merck) was used to hydrolyze the crude protein. Crude protein solution was adjusted to pH=7.5 and equilibrated for 30 min at 37 °C. Protein hydrolysis was performed using a freshly prepared trypsin enzyme solution with enzyme-substrate ratio of 100 U/mg algae. Hydrolysis took place for 4 h under shaking at 180 rpm. The enzymatic reaction was terminated by heating the mixture to 80 °C for 20 min. The hydrolysate was clarified by centrifugation (model Kubota 6500; Shimadzu) at 9880*×g* for 20 min at 4 °C, evaporated by an evaporator (CentriVap, Labconco, Kansas, MO, USA) and stored at –20 °C until required for use.

### Determination of the amino acid composition of Synechococcus sp. VDW cell

#### Acid hydrolysis

According to Prakot *et al*. (*11*), dried *Synechococcus* sp. VDW cells (25 mg) were hydrolyzed with 5 mL of 6 M HCl (Sigma-Aldrich, Merck) at 110 °C for 24 h, after which the mixture was agitated mildly. In order to eliminate any air, the tube was rinsed with nitrogen for 1 min followed by heating at 110 °C for 22 h. Subsequently, the reaction was allowed to cool to room temperature. A volume of 10 mL of 2.5 mM L-α-amino-*n*-butyric acid (Sigma-Aldrich, Merck) in 0.1 M HCl as internal standard (Sigma-Aldrich, Merck) was diluted with distilled water to a final volume of 250 mL. A 0.20-µm filter (Whatman, GE Healthcare, Little Chalfont, UK) was used to filter the solution, and a derivatizing agent 6-aminoquinolyl-N-hydroxysuccinimidyl carbamate (AccQ-Fluor reagent; Sigma-Aldrich, Merck) was added to the filtrate. In order to enable the transformation of the minor tyrosine side product to a main monoderivatized compound, a heating block was utilized to heat the resulting mixture at 55 °C for 10 min.

#### Chromatographic conditions

Liquid chromatography was carried out to separate the analytes in a Waters Alliance 2695 separation module (Milford, MA, USA) with a Hypersil GOLD C_18_ column (50 mm×0.5 mm, 5 µm; Thermo Fisher Scientific, San Jose, CA, USA) and a 5 µm C_18_ Hypersil GOLD guard column (30 mm×0.5 mm; Thermo Fisher Scientific). An aqueous sodium acetate buffer solution (pH=4.90) and 60% acetonitrile (ACN; Sigma-Aldrich, Merck) were then used to remove them isocratically at a flow rate of 0.3 mL/min. The injection volume was 5 µL, while total run time was 15 min.

### Determination of antioxidant activity by DPPH and ABTS radical scavenging assays

#### DPPH radical scavenging activity assay

The DPPH (2,2’-diphenyl-1-picrylhydrazyl) radical scavenging activity was investigated according to the method described by Tanzadehpanah *et al*. (*12*) with slight modification. First, 0.004 g of DPPH (Sigma-Aldrich, Merck) was dissolved in 100 mL of methanol to prepare a 100-µM DPPH radical solution and then 320 µL of this solution were added to 80 µL of the sample. After that, the mixture was incubated in the dark at room temperature for 15 min. Next, 100 µL of each solution were placed into 96-well plates, and the absorbance was read at 517 nm with a microplate reader (model Multiskan GO; Thermo Fisher Scientific Inc.). For the positive control, 0.1 mg/mL of ascorbic acid (Sigma-Aldrich, Merck) was used. The percentage inhibition of DPPH radical scavenging was calculated from the following equation:

FTB-57-358-e1.eps

where *A*_control_ is the absorbance of the solution without the sample, *A*_blank_ is the absorbance of the deionized water, *A*_sample_ is the absorbance of the *Synechococcus* sp. VDW hydrolysate (protein sample), and *A*_background_ is the absorbance of the sample without the DPPH. The IC_50_ values (*i.e*. the concentration of *Synechococcus* sp. VDW hydrolysate required to inhibit the antioxidant activity by 50%) were calculated using the GraphPad Prism software v. 6.01 (*13*). All of these tests were performed in triplicate, and the values are expressed as the mean value±standard deviation (S.D.).

#### ABTS radical scavenging activity assay

The ABTS (2,2’-azino-bis(3-ethylbenzothiazoline-6-sulphonic acid)) radical scavenging activity was investigated according to the method described by Cai *et al*. (*14*) with slight modification. The ABTS cation radical was generated by mixing equal volumes of 7 mM ABTS solution (Sigma-Aldrich, Merck) and 2.45 mM potassium persulfate (Sigma-Aldrich, Merck) in the dark at room temperature for 12 h. The ABTS cation radical solution was diluted to achieve an absorbance at 734 nm of (0.7±0.02) and then 750 µL of the solution were mixed with 25 µL of sample and incubated in the dark at room temperature for 10 min before the *A*_734 nm_ was measured using a microplate reader (model Multiskan GO; Thermo Fisher Scientific Inc.). For the positive control, 0.1 mg/mL of ascorbic acid was used. The percentage inhibition of ABTS radical scavenging was calculated using Eq. 1.

### Protein content determination

Bovine serum albumin (Sigma-Aldrich, Merck) as the standard was used to determine the protein content according to Bradford’s procedure (*15*) at concentrations from 5 to 20 µg/mL to make the calibration curve and the absorbance of the supernatant was measured at 595 nm.

### Peptide enrichment by ultrafiltration and RP-HPLC fractionation

#### Ultrafiltration

The hydrolysate solution was fractionated through a range of nominal molecular mass cut-off membranes of 10, 5 and 3 kDa (Pellicon XL Filter; Merck Millipore, Billerica, MA, USA) to yield fractions with a *M* range of <3.0 kDa (F_A_), 3–5 kDa (F_B_), 5–10 kDa (F_C_) and >10 kDa (F_D_). The obtained protein hydrolysate fractions were stored at –20 °C until further use.

#### Reversed phase (RP-HPLC)

The fraction that had the highest antioxidant activity, F_A_, was filtered through 0.45-µm filters (Whatman, GE Healthcare). RP-HPLC analysis was done using a Spectra System^TM^ HPLC (Thermo Fisher Scientific Inc.), with a reversed phase C_18_ column (250 mm×4.6 mm, Luna 5 µM; Phenomenex, Torrance, CA, USA). Peptides were eluted with a discontinuous gradient comprising mobile phase A (0.1%, *V*/*V*, trifluoroacetic acid (TFA) in distilled deionized water) and mobile phase B (70%, *V*/*V*, ACN in 0.05%, *V*/*V*, TFA) at a flow rate of 0.7 mL/min. TFA and ACN were purchased from Sigma-Aldrich, Merck. The gradient started at A:B=100:0 (*V*/*V*), changing linearly to A:B=90:10 (*V*/*V*) 14 mL, then to A:B=70:30 (*V*/*V*) 8.75 mL and finally to A:B=60:40 (*V*/*V*) 6 mL. Peptides were detected by measuring the absorbance at 280 nm (*A*_280 nm_). Chromatographic analyses were done by ChromQuest software, v. 5.0 (*16*). Four subfractions, F_1_, F_2_, F_3_ and F_4_, were obtained, lyophilized and their antioxidant activities were determined by DPPH and ABTS radical scavenging assays.

### Identification of peptides by quadrupole-time-of-flight mass spectrometry (Q-TOF-MS/MS)

The peptide fraction isolated from RP-HPLC that showed the highest antioxidant activity (F_4_) was subjected to Q-TOF- -MS/MS coupled with electrospray ionization (ESI; model Amazon SL, Bruker, Germany) in order to sequence the main components *de novo*. The MS/MS data were submitted for a database search against the Swiss-Prot database using the Mascot package (*17*).

### Comparison of the antioxidant activity of synthetic peptides and the F_4_ fraction from the RP-HPLC fractionated enzymatic peptides

The peptide sequences obtained from the ESI-Q-TOF-MS/MS analysis of the RP-HPLC fraction F_4_ were synthesized by a Fmoc solid-phase method using peptide synthesizer (model ABI 433A; Applied Biosystems, Foster City, CA, USA). The purity of the peptides was verified by analytical mass spectrometry (model Finnigan™ LXQ™; Thermo Fisher Scientific Inc.) coupled to a Surveyor HPLC. The antioxidant activities of the peptides were determined using the DPPH and ABTS radical scavenging assays.

### Antiproliferation/cytotoxicity activity of the F_A_ peptide hydrolysate fraction

The F_A_ peptide hydrolysate fraction, which had the highest antioxidant activity, was assessed for its *in vitro* antiproliferative/cytotoxic activity on one non-transformed (WI- -38) and five cancer-derived transformed (BT474, CHAGO-K1, HEP-G2, KATO-III and SW620) human cell lines. Five cell lines were obtained from the Institute of Biotechnology and Genetic Engineering, Chulalongkorn University, Thailand. The cell suspensions in complete medium (CM; RPMI with 10% (*V*/*V*) foetal calf serum (FCS; Biochrom Ltd., Cambridge, UK)) were diluted and plated at 200 µL/well into 96-well plates to a final value of *A*_540 nm_=1.0 (about 5·10^3^ cells/well for HEP-G2 and SW620, and 10^4^ cells/well for BT474, CHAGO-K1, KATO-III and WI-38 cells), and then incubated at 37 °C with 5% (*V*/*V*) CO_2_ for 24 h. The cell culture medium was then replaced with fresh CM containing the F_A_ fraction at various concentrations and incubated as described above for 72 h. Next, 10 µL of 5 mg/mL 3-(4,5-dimethylthiazol-2-yl)-2,5-diphenyltetrazolium bromide (MTT; Sigma-Aldrich, Merck) in normal saline solution (Sigma-Aldrich, Merck) were added to each well, mixed and incubated as above for 4 h. The medium was then removed and 150 µL/well of dimethyl sulfoxide (Sigma-Aldrich, Merck) were added to dissolve the insoluble purple formazan before measuring the *A*_540 nm_ with a microplate reader (model Multiskan GO; Thermo Fisher Scientific Inc.). The *A*_540 nm_ was directly proportional to the number of viable cells, so the relative percentage cell viability was calculated from the following:

Cell survival=(*A*_sample_/*A*_control_)·100 /2/

where the control (no sample) was set to be 100% cell survival. The IC_50_ value was determined from the data using GraphPad Prism software v. 6.01 (*13*). All assays were performed in triplicate.

### Apoptosis

Apoptosis was determined by dual staining of the cells with Annexin V-FITC and propidium iodide (PI) following the Annexin V-FITC/PI detection kit protocol (BioLegend Inc., San Diego, CA, USA). The Annexin V-FITC/PI detection kit was used to label phosphatidylserine externalization on the outer plasma membranes, which occurs in early apoptotic cells, while PI staining of DNA indicates cell membrane deficiency and quantitates the cellular DNA content. Apoptosis and necrosis were monitored using quadrant statistics for the various states as follows: viable (Annexin-/PI-), early apoptotic (Annexin+/PI-), late apoptotic (Annexin+/PI+) and necrotic (Annexin-/PI+). The SW620 cells were seeded in 25 cm^2^ culture flasks (10^7^ cells/flask) in CM containing 2.0 mM l-glutamine and incubated overnight. Then F_A_ fraction was added to the cells and incubated for a further 24, 48 or 72 h at 37 °C. The cells were harvested using a scraper, washed with cold phosphate buffered saline (PBS; pH=7.2) containing 1% (*V*/*V*) FCS and centrifuged using centrifuge model Mikro 185 (Hettich, Tuttilingen, Germany) at 13 000*×g* for 15 min at 4 °C. The cell pellets were resuspended in Annexin V-binding buffer (100 µL), and 100 µL of the cell suspension was placed into a 1.5-mL microcentrifuge tube followed by the addition of 2.5 µL of Annexin V-FITC and 5 µL of PI solution. The cell suspension was vortexed and incubated in the dark for 15 min at room temperature, before 200 µL of Annexin V-binding buffer was then added. Apoptosis was immediately detected by flow cytometry (BD FACSCalibur, BD Biosciences, Singapore), and the data were analysed using the FlowJo software, v. 10.6.1 (*18*).

### Caspase-3, -8 and -9 activities

#### Preparation of SW620 cell lysates

The SW620 cells were seeded at 10^7^ cells/flask, and the F_A_ fraction was added at the concentration representing the IC_20_ value for these cells (or 0 µg/mL for the control), and incubated at 37 °C in 5% (*V*/*V*) CO_2_ for 24, 48 or 72 h. The control and treated cells were taken using a scraper and cleaned with 20 mM cold PBS, centrifuged using centrifuge model Mikro 185 (Hettich) at 600*×g* for 15 min, and then the cell pellets were resuspended in 1× lysis buffer (100 µL; Merck, Darmstadt, Germany) and incubated on ice for 15–20 min. The lysed cells were centrifuged at 20 000*×g* for 15 min at 4 °C, and the supernatant (lysate) was moved to a new tube. The lysate was examined immediately or stored at –70 °C until examination.

#### Measurement of caspase-3, -8 and -9 activities

A colorimetric caspase-3, -8 or -9 assay kit (Merck, Darmstadt, Germany) was used to determine the caspase-3, -8 or -9 activity, respectively, depending on the hydrolysis of the acetyl--Asp-Glu-Val-Asp *p*-nitroaniline (Ac-DEVD-*p*NA), acetyl-Ile-Glu-Thr-Asp *p*-nitroaniline (Ac-IETD-*p*NA) or LEHD-*p*NA peptide substrate, respectively, to release the *p*NA moiety. The release of *p*NA was determined from the absorbance at 405 nm. The cell lysates and respective caspase-3, -8 or -9 positive control (5, 10 and 10 µL for caspase-3, -8 and -9, respectively) were loaded into 96-well plates, and 85 or 80 µL of 1× assay buffer for caspase-3 or -8, respectively, or 20 µL of 5× buffer for caspase-9 were then added. The reaction was initiated by loading 10 µL of the respective caspase substrate into each well, mixing gently and incubating at 37 °C for 70–90 min. The *A*_405 nm_ values were converted to *p*NA concentrations using a *p*NA calibration curve and the respective caspase-3, -8 and -9 activity (in µmol/(min·mL)) was then calculated from the following equation:

Activity=(*c*(*p*NA)·df)·(*t*·*V*) /3/

where *V* is the volume of sample (in mL), df is the dilution factor and *t* is the reaction time (in min).

### Statistical analysis

Each determination was performed in triplicate and presented as the mean value±S.D. One-way analysis of variance and Duncan’s multiple range test served to perform all statistical analyses using the Statistical Package for Social Sciences (SPSS) v. 15.0 (*19*). Significance was accepted at p≤0.05.

## RESULTS AND DISCUSSION

### Screening of crude protein hydrolysate for antioxidant activity and its amino acid composition

The age of microalgae *Synechococcus* sp. VDW played a role in determining the preliminary conditions for the screening of antioxidant activity using the DPPH and ABTS radical scavenging activities as targets. It had the largest effect on the antioxidant activity (Fig. S1), where significantly higher DPPH and ABTS radical scavenging activities ((151.2±12.1) and (56.9±0.8) µg/mL, respectively) were found on day 21 than on days 7 or 14. On day 21 of cultivation, *Synechococcus* sp. VDW was in stationary phase, which is in accordance with the observation that microalgae produce secondary metabolites during the stationary phase (*20*–*23*).

The amino acid composition of *Synechococcus* sp. VDW (Table S1) indicates that this strain contains important amino acids that affect the antioxidant activity, namely hydrophobic, aromatic and imidazole amino acids, such as leucine (Leu), methionine (Met), valine (Val), cysteine (Cys), proline (Pro) phenylalanine (Phe), tyrosine (Tyr), tryptophan (Trp) and histidine (His) (*24*, *25*). The dominant essential amino acids in this strain were Leu, Phe and Val, which accounted for 0.17, 0.16 and 0.12% (*m*/*m*), respectively, of the total amino acids. Previous studies have shown that protein hydrolysates from other organisms, including oyster (*26*), sardinelle (*27*), purple sweet potato (*28*) and *Chlorella vulgaris* (*29*), have antioxidant activities. Therefore, peptides derived from *Synechococcus* sp. VDW may have antioxidant activity.

### DPPH and ABTS radical scavenging activities after size fractionation of the crude protein hydrolysate by ultrafiltration

The *Synechococcus* sp. VDW cells were grown for 21 days and hydrolyzed with trypsin to yield the crude protein hydrolysate, which was then fractionated by ultrafiltration with molecular mass cut-off membranes of 10, 5 and 3 kDa to yield the four size fractions F_A–D_. The smallest size fraction, F_A_, clearly had the highest antioxidant activity compared to the other fractions (Table S2), with an IC_50_ of (13.6±0.2) and (11.5±0.3) µg/mL for the DPPH and ABTS radical scavenging activities, respectively, but was still lower than that for ascorbic acid, the positive control, with IC_50_ values of (1.1±0.3) and (127.0±4.3) µg/mL, respectively. Nevertheless, the results suggest that peptides with low molecular mass may exhibit greater antioxidant activity than peptides with high molecular mass, which is in accordance with previous studies. For example, the low-molecular-mass (<0.3 kDa) protein hydrolysate from Alcalase™-digested sweet potato had a higher antioxidant activity than the peptides with higher molecular mass (3–5, 5–10 and >10 kDa), according to the OH-scavenging activity and Fe^2+^-chelating ability (*28*).

### DPPH and ABTS radical scavenging activity of the F_A_ fraction after HPLC fractionation (F_1–4_)

**Fig. 1:**
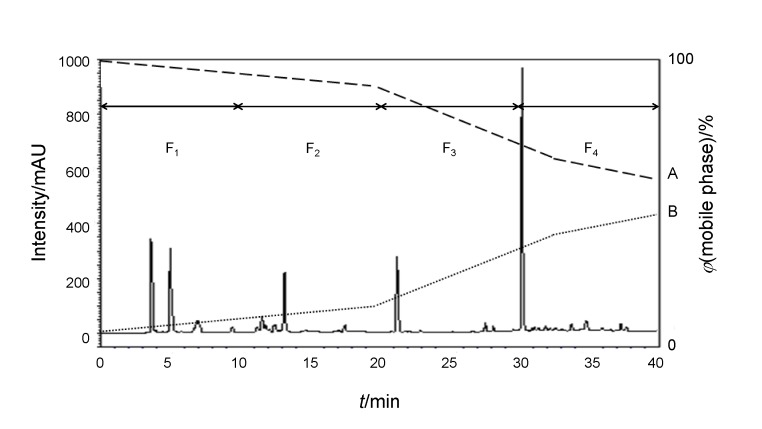
RP-HPLC chromatogram of the F_A_ fraction of the crude *Synechococcus* sp. VDW protein hydrolysate showing the derived subfractions F_1–4_

Fig. 1 shows the chromatogram of the obtained peptides (as *A*_280 nm_ values). Five principal peaks with many minor ones are observable, and these were collected as four subfractions: 0–10 min (F_1_), 10–20 min (F_2_), 20–30 min (F_3_) and 30–40 min (F_4_). The F_4_ subfraction, which had the maximum ABTS radical scavenging activity (Table S3), was selected for further analysis by ESI-Q-TOF-MS/MS.

### Identification of peptides and comparison of the antioxidant activities between the synthetic and enzymatic peptides

The amino acid sequences of five peptides were obtained: Ala-Ile-Leu-Glu-Ser-Tyr-Ser-Ala-Gly-Lys-Thr-Lys (AILESYSAGKTK; 1.27 kDa), Ala-Leu-Asp-Lys-Thr-His-Leu-Ile-Glu-Thr-Lys (ALNKTHLIETK; 1.27 kDa), Ile-Pro-Asp-Ala-His-Pro-Val-Lys (IPDAHPVK; 0.88 kDa), Leu-Leu-Val-His-Ala-Pro-Leu-Lys (LLVHAPLK; 0.88 kDa) and Val-Val-Val-Leu-Arg-Asp-Gly-Ala-Val-Glu-Glu-Leu-Gly-Thr-Pro-Arg (VVVLRDGAVEELGTPR; 1.71 kDa). These five peptide sequences were then commercially synthesized and their antioxidant activity was evaluated in the ABTS and DPPH free radical scavenging assays in parallel with the F_4_ subfraction, from which they were derived. Table 1 summarizes the antioxidant activity (%) of the five synthesized antioxidant peptides. Only AILESYSAGKTK peptide had a high ABTS radical scavenging activity, but its DPPH radical scavenging activity was low. ALNKTHLIETK peptide showed the highest DPPH radical scavenging activity. Sadat *et al*. (*30*) reported that peptides obtained from α-lactalbumin containing mainly Tyr or Trp in the sequence showed the highest ABTS radical scavenging activity. Thus, the relatively higher ABTS radical scavenging activity of the AILESYSAGKTK peptide might be attributed to the Tyr residue in the sequence (*24*, *25*).

**Table 1:** Antioxidant activity of the five synthesized peptides at *γ*=1 mg/mL

Peptide sequence	Formula	*M*/kDa	Purity/%	DPPH scavenging activity/%	ABTS scavenging activity/%
AILESYSAGKTK	C_56_H_95_N_15_O_18_	1.27	99.2	(16.6±0.1)^b^	(46.9±0.1)^a^
ALNKTHLIETK	C_56_H_99_N_17_O_16_	1.27	99.1	(30.9±0.6)^a^	(4.2±0.4)^c^
IPDAHPVK	C_40_H_65_N_11_O_11_	0.88	94.8	(7.7±0.7)^c^	(1.8±1.0)^d^
LLVHAPVK	C_42_H_73_N_11_O_9_	0.88	99.3	(6.3±0.4)^c^	(3.78±1.1)^c^
VVVLRDGAVEELGTPR	C_74_H_130_N_24_O_22_	1.71	93.9	(16.2±0.1)^b^	(11.6±1.9)^b^

Data are presented as mean value±S.D. of triplicate independent repeats. Mean values with different letters in superscript within a column are significantly different (p≤0.05; Duncan’s multiple range test)

All fragments were aligned using Swiss-Prot database (*de novo* deducing) (*17*) to find the homologous region. The longest peptide sequence, VVVLRDGAVEELGTPR, revealed 93% (15/16) similarity of the amino acid sequence to the sugar ABC transporter ATP-binding protein (all from *Synechococcus* sp. PCC 6312), and 81% similarity to the alpha/beta hydrolase *Synechococcus* sp. WH 7803. Peptides LLVHAPVK and IPDAHPVK (*m*/*z*=875.55 and 875.48) were similar to a permease and catalase/hydroperoxidase HPI(I), respectively, from *Synechococcus* sp. PCC 6312 and *Synechococcus* sp. WH 5701, respectively, suggesting that this protein could be a member of this transport/signalling protein as well. The remaining peptides (AILESYSAGKTK and ALNKTHLIETK) did not closely match to any part of the obtainable sequences. This peptide was too short to be examined for valid specific conflict sites. Accordingly, it could be replaced in polymorphic regions in a protein derived from divergent subunit proteins.

Several studies have demonstrated that aromatic and hydrophobic amino acids, including Phe, Try, Typ, Phe, His, Cys and Met, had the highest antioxidant activity. Generally, aromatic amino acids in peptides have a very good radical scavenging activity. Their structure allows the scavenging of unpaired electrons or radicals by donating protons, where the imidazole group in His has proton-donating ability (*24*, *27*, *28*). Additionally, the hydrogen donation involving Gly has been reported to have high antioxidant activity. Likewise, the SH group in Cys is a radical scavenger with an independently important antioxidant action owing to its direct interaction with radicals (*31*).

### Antiproliferation activity of the F_A_ fraction

To investigate the cytotoxicity of the F_A_ fraction, we compared human normal Wi38 cells to the human malignant cell lines BT474 (breast), Chago-K1 (lung), Hep-G2 (hepatoma), KATO-III (gastric) and SW620 (colon), using the MTT assay (Table 2). The cells were treated with the F_A_ fraction at different concentrations for 72 h. The result shows that the F_A_ fraction exhibited cytotoxic activity at various concentrations against the cell lines, with IC_50_ values (in µg/mL): 538.3±3.8 (Wi38), 155.1±6.2 (BT474), 164.7±10.7 (Chago-K1), 166.8±19.6 (Hep-G2), 270.1±26.5 (KATO-III) and 106.6±21.5 (SW620). Thus, the F_A_ fraction had an apparent *in vitro* cytotoxicity on the transformed (human cancer-derived) cell lines but this was much (2.0- to 5.4-fold) weaker on the normal (untransformed) Wi38 cells. Several studies have previously reported that some purified peptides exhibit an antiproliferative activity against cancer cell lines as well as antihypertensive, antiangiogenic and antiobesity effects (*26*, *31*, *32*). Since the IC_50_ value of SW620 was the lowest among all the tested cell lines, we used SW620 cells for the determination of apoptosis in subsequent experiments.

**Table 2:** Cytotoxic effect of F_A_ fraction on normal human cell lines and five cancer cell lines

Cell line	IC_50_/(µg/mL)
Wi38 (normal human cells)	(538.3±3.8)^e^
BT474 (breast)	(155.1±6.2)^ab^
Chago-K1 (lung)	(164.7±10.7)^b^
Hep-G2 (hepatoma)	(166.8±19.6)^c^
KATO-III (gastric)	(270.1±26.5)^d^
SW620 (colon)	(106.6±21.5)^a^

Data are presented as mean value+S.D. of three replicates. Different letters in superscript indicate significant differences among the groups according to Duncan’s test (p≤0.05)

### Relationship between the antioxidant activity and the anticancer (cytotoxic) effect

**Fig. 2:**
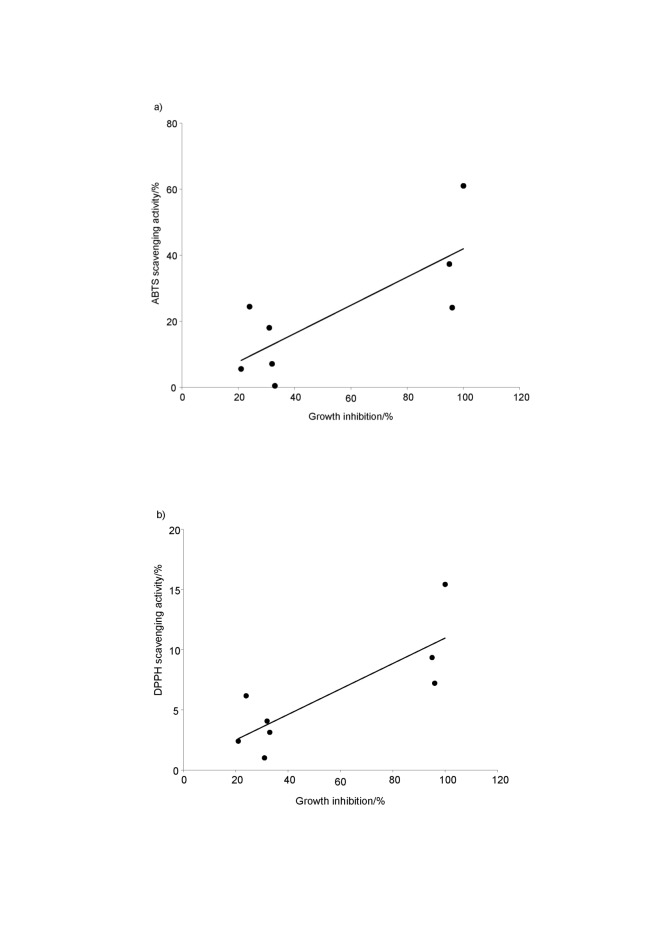
Correlations of the growth inhibition of the SW620 cell line and: a) ABTS and b) DPPH free radical scavenging activities of F_A_ fraction. Data plots are the mean values of three replications

Scatterplots representing relationships between the growth inhibition (%) of SW620 cancer cell lines and the ABTS or DPPH free radical scavenging activities (%) were drawn (Fig. 2). The growth inhibition (%) on SW620 cancer cell lines was positively correlated with the ABTS and DPPH radical scavenging activities of the F_A_ fraction (R^2^=0.6048, p<0.001, R^2^=0.6673, p<0.001, respectively). The potential anticancer activity of the F_A_ fraction (cytotoxicity) could be induced *via* multiple pathways, such as interaction with key enzymes in cellular signalling pathways, cell cycle, apoptosis and metastasis (*32*, *33*). Further studies on the role of the growth inhibitory effect of the antioxidants on the SW620 cancer cell line may lead to the understanding of cancer therapy. So, the knowledge of mechanistic functions between the anticancer activity and the antioxidant efficiency of *Synechococcus* sp. VDW peptides is of great importance.

### Cell apoptotic rate detected by flow cytometry

**Fig. 3:**
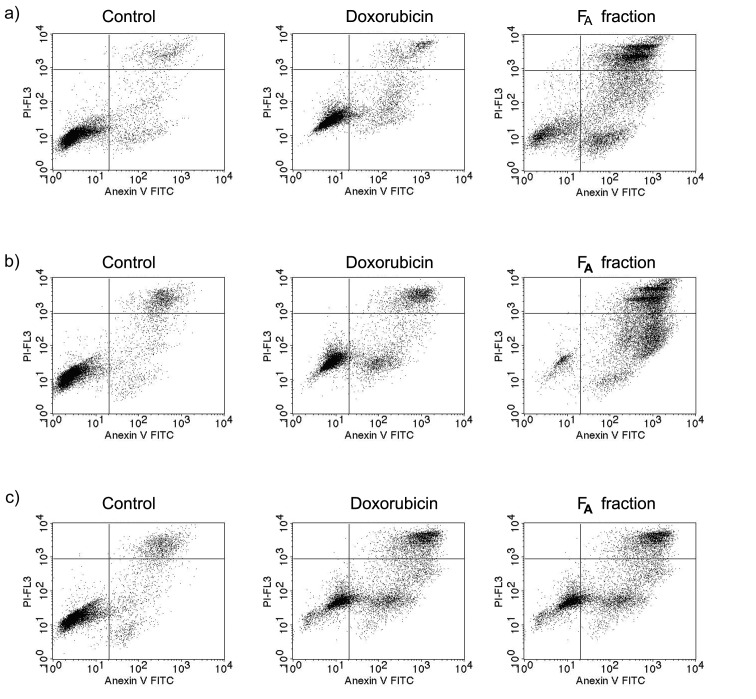
Induction of apoptosis in SW620 cells by F_A_ fraction (IC_50_=23.06 µg/mL) at: a) 24 h, b) 48 h and c) 72 h measured by flow cytometry (upper right quadrant indicates late apoptotic cells, upper left quadrant indicates necrotic cells, lower left quadrant indicates viable cells, and lower right quadrant indicates early apoptotic cells) compared to negative control (no F_A_) and positive control (*γ*(doxorubicin)=0.5 µg/mL). Plots are based on events (cells) and representative of those seen from three independent repeats

Apoptosis is the process of programmed cell death that occurs in multicellular organisms when cells are damaged by disease or toxic agents. Apoptotic cells were detected using Annexin V FITC in tissues by flow cytometry. The SW620 cells were treated with the F_A_ fraction (IC_50_ value of 23.06 µg/mL) to detect apoptosis, as shown in Fig. 3.

The SW620 cells treated with 23.06 µg/mL F_A_ showed increasing percentages of early apoptotic cells over time, with values of 32.11, 48.10 and 52.24% at 24, 48 and 72 h, respectively (Table 3). The percentage of cells in total apoptosis was 79.86, 95.85 and 97.95% at 24, 48 and 72 h, respectively (Table 3). In the control group, the total apoptosis was 15.44, 16.77 and 24.72% at 24, 48 and 72 h, respectively. The percentage of cells in early apoptosis following F_A_ treatment for 48 h was similar to the treatment for 72 h but was higher than the treatment for 24 h and the one with doxorubicin (0.5 µg/mL) at all three time points. The proportion of cells in total apoptosis was likely to be affected the most in early apoptosis due to the presence of intrinsic inducers of apoptosis, even in the control cells. This is due to the cultured cells being under stress conditions, such as heat, chemotherapeutic agents, oxidative stress, irradiation and nutrient deficiency, which all serve as stimuli to induce the apoptotic process (*34*, *35*). In a previous study (*36*), the scorpion venom peptide BmKn2 was used to determine the mechanism of induction of apoptosis on oral cancer cells. Furthermore, the BmKn2 peptide has an effect on induction of apoptosis in oral cancer cells *via* a p53-dependent intrinsic pathway. The flow cytometry was used for detection of cell apoptosis in human cervical cancer cells. These cells were treated for 6 h with 128 µg/mL ZXR-1 peptide (FKIGGFIKKLWRSKLA), which has the ability to induce cell apoptosis. The early apoptosis value was 55.1%, while the control group showed the value of 2.47%, which was lower than the cells treated with ZXR-1 peptide. The late apoptosis of the cells treated with ZXR-1 peptide and the control was 5.8 and 0.3%, respectively. Moreover, the ZXR-1 peptide proved that it could induce cell apoptosis by targeting mitochondrial membrane to release apoptotic factors by the intrinsic pathway (*37*).

**Table 3:** The proportion of early and total apoptotic SW620 cells as analysed by flow cytometry

Apoptotic activity	*t*/h		
24	48	72
Early	*N*(cell)/%		
Control	(10.51±2.50)^ab^	(7.57±0.69)^d^	(9.72±2.50)^ab^
Doxorubicin	(13.56±0.41)^b^	(19.13±2.24)^c^	(29.03±4.21)^a^
F_A_ fraction	(32.11±1.16)^d^	(48.10±3.46)^e^	(52.24±1.09)^e^
Total			
Control	(15.44±2.42)^a^	(16.77±3.14)^ab^	(24.72±7.58)^b^
Doxorubicin	(20.22±1.33)^ab^	(36.62±4.11)^c^	(63.18±2.64)^a^
F_A_ fraction	(79.86±1.24)^e^	(95.85±2.46)^f^	(97.95±1.35)^f^

Data are presented as mean value±S.D. of three replicates. Mean values with a different letter in superscript are significantly different (p≤0.05; Duncan's multiple range test)

Apoptotic cell death occurs through intrinsic and extrinsic pathways. The intrinsic (mitochondrial) pathway is initiated by the upregulation of wild-type p53 and involves the transcriptional or post-transcriptional regulation of Bcl-2 proteins, and the release of cytochrome c from the mitochondrial intermembrane spaces, thereby activating executioner caspases. The extrinsic (cell surface death receptor) pathway is activated by death receptor ligation, adaptor recruitment, procaspase-8 recruitment, caspase-8 activation and activation of executioner caspases. Additionally, the intrinsic and extrinsic pathways are linked *via* BID cleavage (*38*). In addition, necrosis is another cell death process. It has long been regarded as an unprogrammed death of cells and living tissues, which is considered the primary form of cell death caused by inflammation. Apoptosis eliminates cells during development and homeostasis in tissues, but is important for the disposal of cancer cells that are damaged. Indeed, apoptosis is a necessary process in cancer cells (*39*).

This study suggested that the F_A_ fraction from *Synechococcus* sp. VDW cells induces the apoptotic pathway in SW620 colon cancer cells. Several studies have concluded that purified peptides induce apoptosis, as determined by flow cytometric analysis (*40*). The anticancer effect of *Angelica dahurica* extract was reported in HT-29 colon cancer cell lines, where increasing concentrations of the extract resulted in an increased percentage of cells in early and total apoptosis after treatment for 48 h (*41*).

### Caspase-3, -8 and -9 activation in cancer cell line

The F_A_ fraction with an IC_50_ value of 23.06 µg/mL was used to treat the SW620 cells for 24, 48 and 72 h to investigate the caspase-3, -8 and -9 activities, as determined by colorimetric assays (Table 4). The F_A_ fraction induced the activities of caspase-3, -8 and -9. The highest caspase activity was after 72 h, confirming that F_A_ fraction induced apoptosis *via* activation of caspases-3, -8 and -9, and so activated both the intrinsic and extrinsic pathways of apoptosis (*32*, *41*). The intrinsic pathway is implicated by the increased caspase-9 activity, which was detected at all time points, with the highest activity exhibited at 72 h. Stress conditions activate caspase-9 in cancer cell lines (*37*). The F_A_ fraction presumably induces caspase-9 *via* the mitochondrial pathway like ginsenoside in MG-63 cells. The use of the extrinsic pathway was implicated by the increased caspase-3 and -8 activities, implying that this process was necessary to involve the death receptor-mediated apoptotic pathway (*42*). Caspases-3 and -8 are important enzymes that control programmed cell death (*19*). Caspase-3 is a central caspase, and it plays a key role in the apoptosis pathway and is used to investigate apoptosis (*43*).

**Table 4:** Caspase-3, -8 and -9 activities in SW620 cells treated with F_A_ fraction for 24–72 h

*t/*h	Activity/(µmol/(min·mL)		
Caspase-3	Caspase-8	Caspase-9
Control	(10±7)^c^	(60±22)^c^	(1421±434)^d^
24	(30±19)^c^	(70±44)^c^	(1800±164)^c^
48	(100±7)^b^	(500±68)^b^	(10516±914)^b^
72	(160±7)^a^	(620±66)^a^	(16105±729)^a^

Data are presented as mean value±S.D. of triplicate independent repeats. Mean values with a different letter in superscript within a column are significantly different (p≤0.05; Duncan’s multiple range test)

Chen *et al*. (*44*) reported that baicalin (200 µM) induced apoptosis in SW620 human colorectal carcinoma cells, as indicated by the increased activities of caspases-3, -8 and -9 as well as suppressed SW620 cell growth. Colon cancer cell (HCT-116) apoptosis was induced by the Dae-Hwang-Mok-Dan-Tang (DHMDT) extract, a traditional Korean medicine, at increasing concentrations of 0 to 1 mg/mL, *via* both the intrinsic and extrinsic pathways as measured using caspase-3, -8 and -9 activities (*42*). Shangguan *et al*. (*45*) reported that the apoptotic process in MG-63 human osteosarcoma cells treated with ginsenoside Rf for 24 h reflected the increased caspase-3 and -9, except caspase-8 activities.

## CONCLUSIONS

Taken together, the results of the present study show that the extract with trypsin obtained from *Synechococcus* sp. VDW using the procedure described here had antioxidant and cytotoxic (potential anticancer) properties. Fractionation by ultrafiltration and gel filtration resulted in a crude peptide fraction (F_4_) with a low molecular mass and high antioxidant activity. The extract prepared from these microalgae efficiently induced apoptosis in human cell lines with the highest activity against SW620 cells. Furthermore, these results suggest that the apoptotic pathway in SW620 cells involved activation of caspases-3, -8 and -9. These findings demonstrate that our preparation obtained from microalgae might be a useful source of natural antioxidant agents and antitumor drugs in the future. We have identified one peptide (AILESYSAGKTK) from this preparation as a potential antioxidant compound.
